# Forkhead containing transcription factor Albino controls tetrapyrrole-based body pigmentation in planarian

**DOI:** 10.1038/celldisc.2016.29

**Published:** 2016-08-02

**Authors:** Chen Wang, Xiao-Shuai Han, Fang-Fang Li, Shuang Huang, Yong-Wen Qin, Xian-Xian Zhao, Qing Jing

**Affiliations:** 1 Key Laboratory of Stem Cell Biology, Institute of Health Sciences, Shanghai Jiao Tong University School of Medicine & Shanghai Institutes for Biological Sciences, Chinese Academy of Sciences, Shanghai, China; 2 Department of Cardiology, Changhai Hospital, Shanghai, China

**Keywords:** body color, FoxP, *PBGD*, pigmentation, planarian, tetrapyrrole

## Abstract

Pigmentation processes occur from invertebrates to mammals. Owing to the complexity of the pigmentary system, *in vivo* animal models for pigmentation study are limited. Planarians are capable of regenerating any missing part including the dark-brown pigments, providing a promising model for pigmentation study. However, the molecular mechanism of planarian body pigmentation is poorly understood. We found in an RNA interference screen that a forkhead containing transcription factor, *Albino*, was required for pigmentation without affecting survival or other regeneration processes. In addition, the body color recovered after termination of *Albino* double stranded RNA feeding owing to the robust stem cell system. Further expression analysis revealed a spatial and temporal correlation between *Albino* and pigmentation process. Gene expression arrays revealed that the expression of three tetrapyrrole biosynthesis enzymes, *ALAD, ALAS* and *PBGD*, was impaired upon *Albino* RNA interference. RNA interference of *PBGD* led to a similar albinism phenotype caused by *Albino* RNA interference. Moreover, *PBGD* was specifically expressed in pigment cells and can serve as a pigment cell molecular marker. Our results revealed that *Albino* controls planarian body color pigmentation dominantly via regulating tetrapyrrole biogenesis. These results identified *Albino* as the key regulator of the tetrapyrrole-based planarian body pigmentation, suggesting a role of *Albino* during stem cell-pigment cell fate decision and provided new insights into porphyria pathogenesis.

## Introduction

Biological pigments provide basic granules to form overall body color of an organism as well as critical compartments for cytochrome within a single cell. A comprehensive understanding of the pigmentation process offers us a theoretical foundation for treatments of pigment-related disorders and especially for stem cell-based regenerative medicine. In this case, an *in vivo* model for pigmentation study is of great importance. Owing to possessing powerful regenerative abilities, planarian serves as a distinctive model for stem cell and regeneration studies [1, 2]. Neoblasts, the planarian adult stem cells, respond to injury and then proliferate and differentiate into corresponding cells required for complete regeneration including pigment cells [3–6]. A cell subpopulation among the neoblasts called cNeoblasts is pluripotent and single cell transplantation of cNeoblasts is able to rescue lethally irradiated planarian [7]. Pigmentary system derived from adult stem cell *in vivo* is a unique model for pigmentation study. These features confer planarian to be an advantageous model for pigmentation study. However, regulation of the pigmentation process, especially body pigmentation in planarian, is poorly understood.

Transcription factors have critical roles in guiding the regeneration processes [8, 9]. Vital transcription factors have been identified in planarian polarity establishment [10–14], nerve system [15–17], eye [18–20], pharynx [21, 22], germline [23–25], gut [7, 26] and nephridia [27] development and regeneration. Among these, forkhead containing transcription factors FoxD and FoxA independently regulate anterior pole establishment and pharynx regeneration, indicating that Fox family genes show significant roles during planarian development and regeneration relevant to their functions in mammals [11, 12, 21, 22, 28]. Still, the role that transcription factors have in planarian body pigmentation remains unknown.

Planarian eyes contain pigment cells that utilize melanin as photosensitive pigments and *Gtso*, *Djsix-1*,*Djeya*,*Smed-sp6–9*,*Smed-dlx* and *Smed-ovo* are reported to be critical factors for the generation of eye pigment cup cells in which melanin is synthesized [18–20, 29]. However, the pigments that form planarian body color require further investigations. Krugelis-Macrae [30] suggested the occurrence of porphyrin, a cyclic form of tetrapyrroles in planarian *Dugesia dorotocephala*, by chemical and spectral experiments and demonstrated that planarians are able to convert delta-aminolevuliwic acid to porphobilinogen [31, 32]. This porphyrin-like pigment was further localized in epidermal rhabdites [33], suggesting that the tetrapyrrole-like pigment is responsible for planarian body color. Recently, body pigment cells have been observed by using electron microscopy in *D. gonocephala*, *D. ryukyuensis* and *Schmidtea mediterranea*, whereas chromatograph results suggested that a tryptophan-based pigment ommochrome is responsible for the body color [34–37]. However, molecular or functional validations shall be carried out to confirm these findings and, more importantly, how this process is regulated remains unclear. Tetrapyrroles, referred to as ‘the pigment of life’, are a family of fundamental compounds generated from four porphobilinogen molecules under the catalysis of porphobilinogen deaminase (PBGD) [38–41]. As the intermediate products for heme, chlorophyll and cobalamin, tetrapyrroles typically serve as the colored chromophores. Tetrapyrrole-based biomolecules modulate cellular response to light, oxygen and other environmental factors, whereby tetrapyrrole dysregulation results in serious diseases in human [42, 43]. Whether tetrapyrroles are responsible for planarian body pigments needs further molecular validations.

In this work, we screened for transcription factors that are specifically required for planarian *S. mediterranea* body pigmentation. Our result revealed that a forkhead domain transcription factor is required for body pigmentation without affecting regeneration. We further examined its downstream targets via microarray. Interestingly, this forkhead domain transcription factor controls the expression of enzymes for tetrapyrrole synthesis, and loss of one of these enzymes, *PBGD,* resulted in the same albinism phenotype. Thus, we report a critical transcription factor that controls body color via regulating tetrapyrrole biogenesis in planarian.

## Results

### Planarian body pigment accumulates during juvenile development and regeneration

Newly hatched worms were born colorless and the pigmentation process took place after the hatching, and it took 12 days for a newborn planarian to get pigmented but the eye spots were pigmented at birth (Supplementary Figure S1A). Moreover, the newly generated pigments emerged randomly without a particular pattern, suggesting an irregular growth status and shape of pigment cells (Supplementary Figure S1A). The difference between body color pigmentation and eyespot pigmentation indicates the presence of two separate pigment systems for eyespot and body pigments. Applying the modified transmission electron microscopy (TEM) protocol [35], we confirmed that planarian pigment granules lay between circular and longitudinal muscle cells just beneath the basal lamina (Supplementary Figure S1B), in line with the previous publications [34–37]. Unlike the vital organs of planarian, the body color took more than a fortnight to recover post amputation (Supplementary Figure S1C). The worms regenerated visible eye spots within 6 days, whereas the blastema remained unpigmented. The first visible pigment within the newly regenerated blastema appeared ~8 days post amputation (dpa), and at least 6 more days were indispensable for a full recovery of body color (Supplementary Figure S1C). A schematic diagram showed the pigment cell location: pigment cells locate between the circular and longitudinal muscles beneath the basal lamina (Supplementary Figure S1D). Thus, planarians displayed a different dynamics from the eyespot to generate and regenerate pigment cells and pigments, and the pigmented cells are located between the muscle cells beneath the basal lamina without an orderly pattern.

### *Albino* is required for planarian body pigmentation

**Figure 1:**
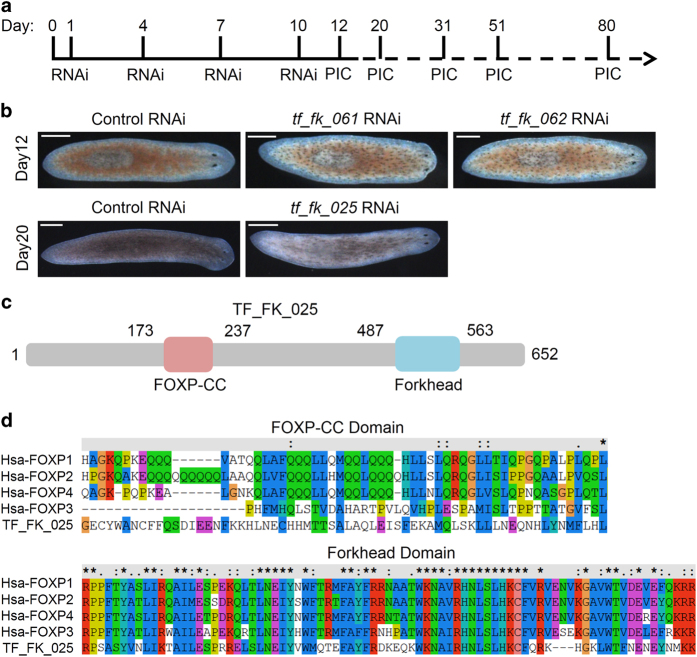
Planarian pigmentation process and transcription factors involved. (**a**) Experimental design of RNAi screen; worms were fed dsRNA-mixed liver on days 1, 4, 7 and 10 for four times and pictures were collected from the twelfth day post first RNAi. (**b**) Genes identified from the RNAi screen that affect the pigmentary system during homeostasis. Scale bar: 200 μm. (**c**) Conserved domain analysis revealed a FOXP-CC domain and a Forkhead domain within protein TF_FK_025. (**d**) ClustX2 analysis of TF_FK_025 with human FOXP family proteins (Star indicates same amino acid).

In order to explore the key factors during the pigmentation process of planarian, we carried out an RNA interference (RNAi) screen against ~600 unreported transcription factors. In the RNAi experiments, we fed the worm with *in vitro* synthesized double stranded RNA (dsRNA) four times within 10 days and observed the phenotype caused by dsRNA feeding (Figure 1a). In the screen, three genes were identified to be required for planarian body color maintenance: RNAi either of *tf_fk_061* or *tf_fk_062* led to pigment clumping and head regression resulting in worm lysis eventually, whereas RNAi of *tf_fk_025* caused albinism without affecting the homeostasis or regeneration (Figure 1b). Using 5ʹ and 3ʹ rapid amplification of complementary DNA (cDNA) ends, a full length of 652 amino acids for *tf_fk_025* was acquired and, using PFAM (Database of protein families. http://pfam.xfam.org/) alignments, a FOXP-CC domain and a Forkhead domain from its amino acids 173 to 237 and from 487 to 563 were identified, respectively (Figure 1c). The FOXP-CC and Forkhead domains were found to be conserved with human FOXP homologs analyzed using ClustalX2 (Figure 1d). As expected, TF_FK_025 was clustered with the human FOXP subfamily (Supplementary Figure S1E). Interestingly, both TF_FK_061 and TF_FK_062 also contain a Forkhead domain (Supplementary Figure S1F). As *tf_fk_025* RNAi leads to a gradual loss of color, we name this planarian FoxP gene *smed-Albino* (henceforth referred to as *Albino*).

### *Albino* is specifically required for pigmentation without affecting worm survival or regeneration

**Figure 2:**
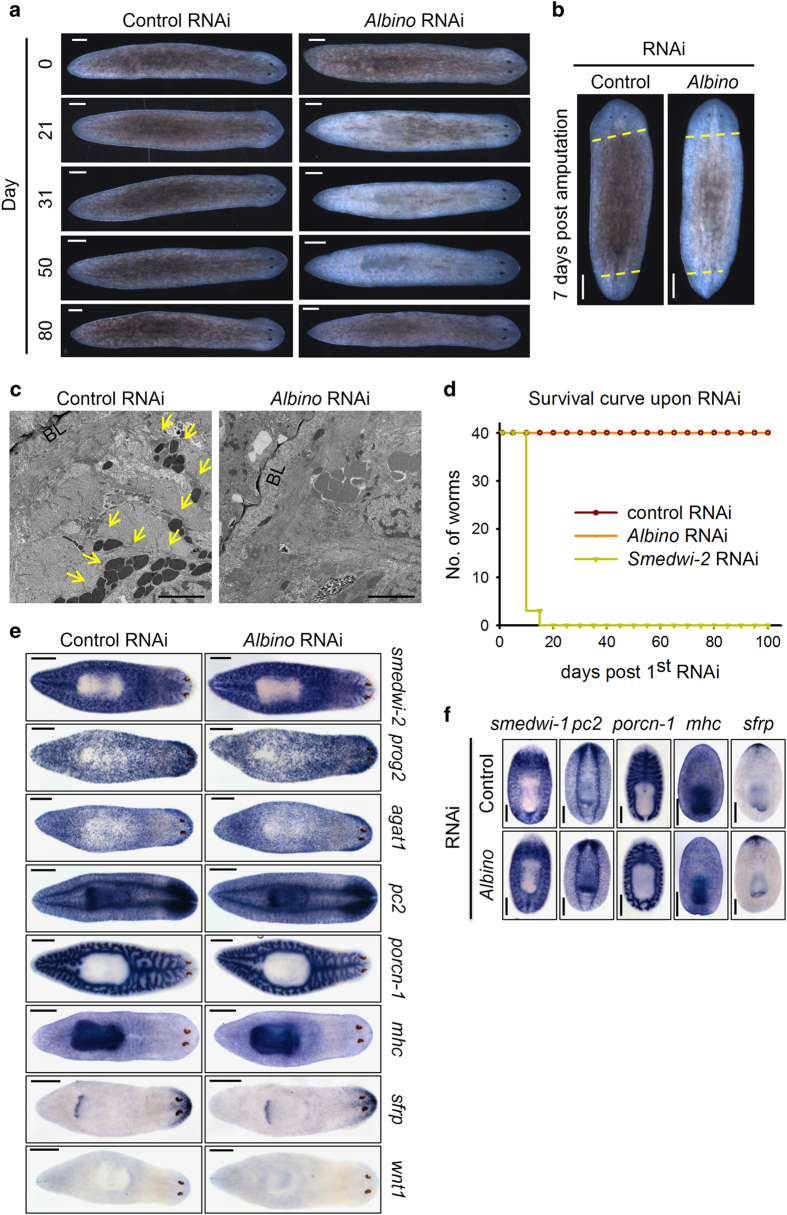
*Albino* is required for pigmentation without affecting survival or regeneration. (**a**) *Albino* RNAi worms lost body color. Scale bar: 200 μm. (**b**) Regenerated *Albino* RNAi trunk fragment with albino phenotype. Scale bar: 200 μm. (**c**) Transmission electron micrographs showing the pigment loss in *Albino* RNAi worms. Scale bar: 20 μm. Yellow arrows indicate pigment granules. (**d**) Survival curve of RNAi worms. (**e**) ISH of stem cell progeny and tissue-specific markers of control and *Albino* RNAi intact planarian. Scale bar: 500 μm. (**f**) ISH of stem cell progeny and tissue-specific markers of control and *Albino* RNAi-amputated trunks. Scale bar: 500 μm.

To gain insight into *Albino* function, we observed the albinism resulted from *Albino* RNAi in detail. *Albino* RNAi worms lost body color gradually from Day 21 post RNAi and became completely albino on around Day 50 post RNAi (Figure 2a and Supplementary Figure S2A). Amputated worms upon *Albino* RNAi successfully regenerated lost head and tail just as the control RNAi worms, but the newly regenerated parts were unpigmented and the original parts became albino as the intact worms (Figure 2b). Transmission electron microscopy confirmed that the pigment granules lost upon *Albino* RNAi, as we observed no pigment granules within the basal lamina where the pigment cells reside (Figure 2c and Supplementary Figure S2B). To ascertain whether *Albino* RNAi affects the worm survival, we fed the worms food containing *Albino* dsRNA for over 100 days and the worms survived with albinism while *smedwi-2* RNAi worms died [44] within 20 days (Figure 2d). *In situ* hybridization (ISH) of specimen 30 days post control or *Albino* RNAi revealed that neither stem cells, nerve system, gut, muscle nor polarity markers were affected (Figure 2e), although the regenerating worms also showed no differences in the expression pattern for makers of stem cells, nerve system, gut, muscle and anterior polarity (Figure 2f). As the stem cell system was unaffected upon *Albino* RNAi, we hypothesized that albino worms would recover as the RNAi effects decrease. As expected, planarian subjected to four rounds of dsRNA feeding and reverted with normal food first became albino and then became re-pigmented around 80 days post first RNAi (Figure 2a). These results indicate that the Forkhead domain containing gene *Albino* is required for the pigmentation process of planarian without affecting the worm survival or regeneration, and the robust stem cell system allows the worms to become re-pigmented once the RNAi is terminated.

### *Albino* enriches in epidermal region and newly regenerated blastema

**Figure 3:**
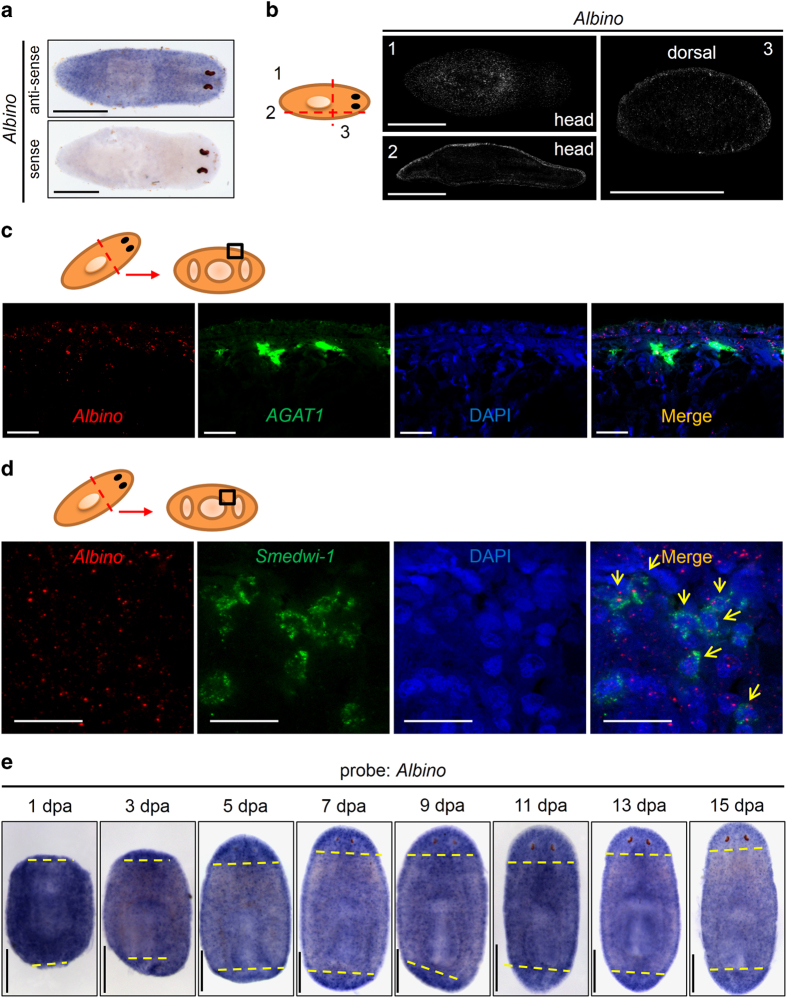
*Albino* enriches at epidermal region and during regenerating blastema. (**a**) WISH in intact animals. Scale bar: 500 μm. (**b**) Vibration sections of FISH animals. Section thickness: 80 μm and scale bar: 500 μm. Images are z-stacks of 10 μm. (**c**) Double FISH with *AGAT1* and *Albino* in wild-type animals showing the dorsal body wall. Scale bar: 20 μm. Cartoon indicates region of interest. Images are single confocal sections. (**d**) Double FISH with *smedwi-1* and *Albino* in wild-type animals showing a transect section. Scale bar: 25 μm. Yellow arrows indicate *smedwi-1* and *Albino* double-positive cells. Images are single confocal sections. (**e**) ISH in regenerating animals detecting *Albino*. Yellow dashes indicate amputation sites. Scale bar: 500 μm.

To understand how *Albino* controls pigmentation, systematic expression analyses were carried out (Figure 3). We first synthesized an antisense probe for *Albino* detection and a sense probe to validate the signals. Whole-mount ISH confirmed the specificity of the signals (Figure 3a). As shown in whole-mount- and section fluorescence *in situ* hybridization (FISH), *Albino* is ubiquitously expressed throughout the body of a whole worm. Strong signals were observed at the surface and edges, indicating superficial expression enrichment (Figure 3b). Vibration sections were performed to gain insight into the detailed expression patterns. We found a clear enrichment of *Albino* around the worms in the epidermal region in both transverse and vertical sections, and *Albino* was also observed to express in mesenchymal tissues (Figure 3b). To further elucidate *Albino* expression patterns, we applied double FISH (DFISH) for *Albino* with *prog2* and *AGAT1*, two stem cell progeny markers known to express at the superficial region of the worms. It was clear in the highly magnified panels that *Albino* expressed in cells beneath epidermal cells as well as in epidermal cells, and colocalized with both *prog2*- and *AGAT1*-positive cells (Figure 3c and Supplementary Figure S3A).

As we observed a mesenchymal-like expression, we next explored the possibility of whether *Albino* expresses in *smedwi-1*-positive adult stem cells. We observed an expression of *Albino* in fluorescence-activated cell-sorting sorted X1 cells with quantitative polymerase chain reaction (qPCR) and a weak reduction of *Albino* signals in γ-ray-irradiated worms using ISH (Supplementary Figure S3B and C). DFISH showed the colocalization of *smedwi-1* with *Albino*, suggesting a role that *Albino* may have in pigment cell fate commitment (Figure 3d). In addition, no defects in food uptake were noted in *Albino* RNAi worms as the worms were fed with food with *Albino* dsRNA for over 100 days (Figure 2d).

Because it took more than 2 weeks to accomplish pigmentation during regeneration, we hypothesized that *Albino* enriches at the unpigmented blastema during regeneration. We observed the expression pattern of *Albino* in wild-type regenerating specimen from the first to fifteenth day of regeneration. As expected, we found an enrichment of *Albino* signals in the newly regenerated areas, suggesting a requirement of this putative transcription factor (Figure 3e). In addition, we found in 2 days' post-hatching worms that *Albino* is not as ubiquitous as in mature planarian according to the fact that the newly hatched worms are not fully pigmented (Figure 1b and Supplementary Figure S3D). These data demonstrated that the *Albino* expression pattern meets the criteria for a pigment cell regulator both spatially and temporally, suggesting a role of *Albino* as a molecular ‘switch’ for pigment generation in planarian.

### Tetrapyrrole pathway enzyme expressions depend on *Albino*

We then used planarian-customized mRNA expression array to elucidate the underlying mechanism. We applied two biological replicas of worms subjected to four rounds of either *Albino* or control RNAi and collected RNA at 7 days post the last RNAi even though the worms were not complete albino. Genes with more than twofold decrease in both replicas are listed in Table 1. Interestingly, the most significantly reduced gene is *PBGD*, which is a critical enzyme in biogenesis of tetrapyrrole. We further noticed in Table 1 that two additional enzymes required fortetrapyrrole biosynthesis, *delta-aminolevulinic acid dehydratase* (*ALAD*) and *5-aminolevulinic acid synthase* (*ALAS*), were also downregulated upon *Albino* RNAi, implying that *Albino* governs planarian pigmentation through tetrapyrrole pigment pathway. Tetrapyrrole comprises four pyrrole rings and is synthesized from glycine and succinyl CoA under the catalysis of ALAS, ALAD and PBGD. If further catalyzed, tetrapyrrole forms, cyclic tetrapyrrole, the cores of natural pigment hemoglobin or chlorophyll [38, 43]. Because of the high degree of conjugation in tetrapyrrole, it is commonly colored as chromophores. Thus, we aligned the planarian amino-acid sequences of ALAD, ALAS and PBGD with the corresponding human and mouse genes, and found that these genes were clustered with their own family with identity of ~50% (Supplementary Figure S4A and B). ClustalX2-aligned results revealed that the amino-acid sequence of *PBGD* shares a high similarity with human and mouse homologs (Supplementary Figure S4C). We thus named these genes *smed-ALAD*, *smed-ALAS* and *smed-PBGD* (henceforth abbreviated to *ALAD*, *ALAS* and *PBGD*, respectively).

**Figure 4:**
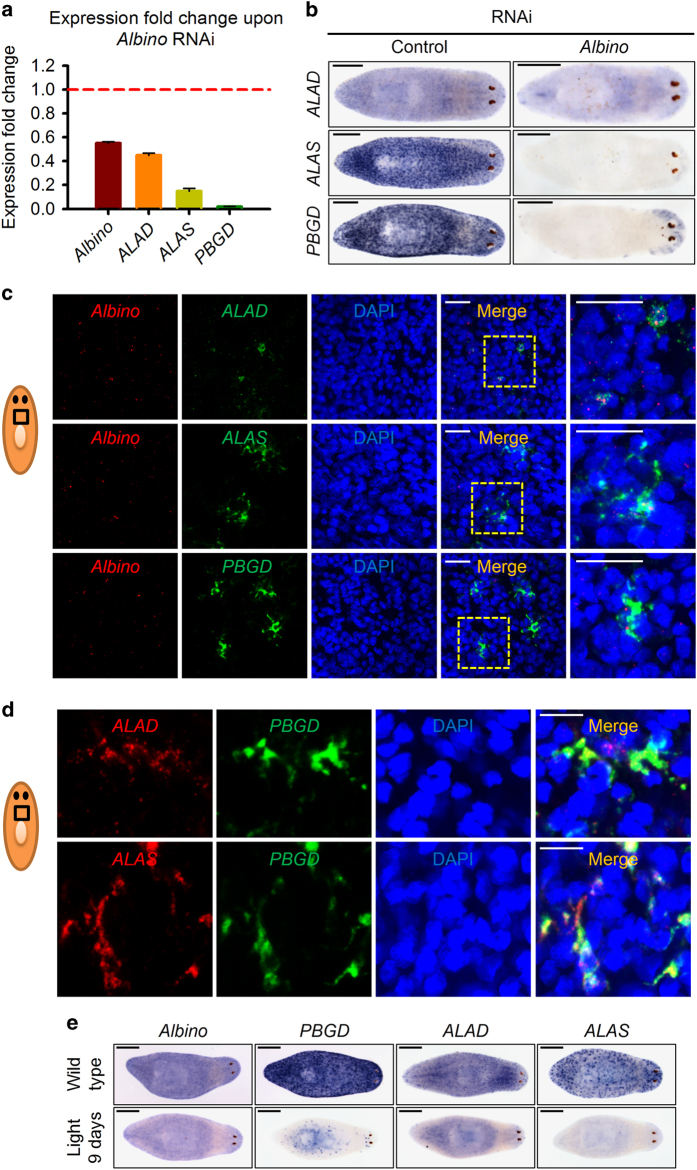
*Albino* regulates expression of tetrapyrrole biosynthetic enzymes in planarian. (**a**) Expression fold changes of tetrapyrrole biosynthetic enzymes upon *Albino* RNAi. Shown are averages of three independent experiments; error bars=s.d. (**b**) WISH for tetrapyrrole biosynthetic enzymes in worms upon control or *Albino* RNAi, indicating the reduction of tetrapyrrole biosynthetic enzyme expressions. WISH samples were collected 7 days post fourth RNAi. Scale bar: 500 μm. (**c**) Representative double FISH results of *ALAD, ALAS* and *PBGD* with *Albino* in wild-type animals. Scale bar: 20 μm. Images are single confocal sections. (**d**) Representative double FISH results of *ALAD* and *ALAS* with *PBGD* in wild-type animals. Scale bar: 20 μm. Images are single confocal sections. (**e**) ISH of either wild-type worms or worms received 9 days of continuous direct light for *Albino*, *PBGD*, *ALAD* and *ALAS*.

The confirming qPCR results showed that the fold changes of *ALAD*, *ALAS* and *PBGD* were identical to those in the mRNA array. Moreover, *PBGD* is notably reduced by more than 50-folds, suggesting that *PBGD* expression is largely dependent on *Albino* (Figure 4a). ISH confirmed the decrease in tetrapyrrole synthesis enzymes and, although with different expression levels, we noticed a similar expression pattern of all three enzymes at superficial layers of worms (Figure 4b). As expected, we found that *Albino* colocalized with these enzymes at superficial regions (Figure 4c). In addition, these enzymes also colocalized with each other as revealed by DFISH at the same region that *Albino* is expressed (Figure 4d).

Tetrapyrrole could be further catalyzed into heme, which is a critical biological pigment required for many cell activities such as electron transfer, catalysis and molecule transport [45]. We next examined the remaining enzymes (*Uroporphyrinogen Decarboxylase*, *UROD*; *Uroporphyrinogen III Synthase*, *URO3S*; *Coproporphyrinogen Oxidase*, *CPOX*; *Protoporphyrinogen Oxidase*, *PPOX*) required for heme biosynthesis. However, no significant changes of these enzymes at the mRNA expression level were observed (Supplementary Figure S5A). Besides, these enzymes exhibited different expression patterns from the tetrapyrrole enzymes (Supplementary Figure S5B), indicating that heme is not involved in the pigmentation process in planarian. We also tested the expression-level changes of stem cell progenies and critical enzymes in melanin and ommochrome pathways and found that no changes over twofold were observed upon *Albino* RNAi in either stem cell progenies or critical enzymes in the melanin pathway (Supplementary Figure S5C). A significant reduction in *kynurenine-3-monooxygenase* (*Kmo*), an important enzyme in the ommochrome pathway, was detected using qPCR (Supplementary Figure S5C) and was subsequently confirmed by ISH (Supplementary Figure S5D). Interestingly, *Albino* colocalized with *Kmo2* (Supplementary Figure S5E), suggesting that this enzyme is partially involved during planarian pigmentation.

Our laboratory noted a phenomenon of light bleaching several years ago: planarian lost body color by excreting the pigmented mass from the pharynx, whereby the worms became unpigmented after 9 days' exposure to continuous light (Supplementary Figure S5F). This result was supported by a recent report [46]. It is significant to understand whether the *Albino*-mediated pigmentary system was affected by this progress. As expected, the expression levels of both *Albino* and tetrapyrrole-related enzymes were decreased in light-bleached worms, further indicating that *Albino*-mediated tetrapyrroles are responsible for the body color of planarian (Figure 4e).

Taken together, our data demonstrate that the expression of tetrapyrrole biosynthesis enzymes depends on the expression of *Albino*. The colocalization of *Albino* and these enzymes provides the basis for a regulatory network between *Albino* and these enzymes. In addition, it is noteworthy that *Albino* is required for the expression of *Kmo2*, an enzyme in ommochrome biosynthesis.

### *PBGD* RNAi resulted in albinism during regeneration and homeostasis

**Figure 5:**
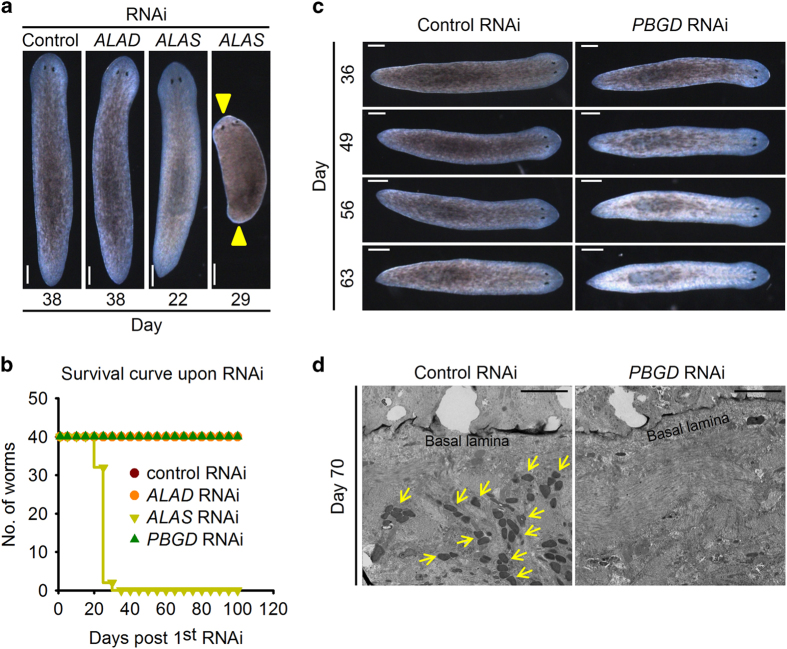
*PBGD* RNAi results in albinism. (**a**) RNAi of tetrapyrrole biosynthetic enzymes. Yellow arrows indicate regressions. Scale bar: 200 μm. (**b**) Survival curve of RNAi worms. (**c**) *PBGD* RNAi worms lost body color gradually and got totally albino ~60 days post first RNAi. (**d**) Transmission electron micrographs showing the pigment lost in *PBGD* RNAi worms. Scale bar: 20 μm. Yellow arrows indicate pigment granules.

RNAi experiments of these enzymes were carried out in order to elucidate their functions in planarian pigmentation. RNAi of *ALAD* caused no significant change in pigmentation, homeostasis or regeneration (Figure 5a and b and Supplementary Figure S6A). Surprisingly, *ALAS* RNAi resulted in a slight body color loss at ~22 days post first RNAi, but a strong defect in homeostasis 7 days later starting with a regression from both head and tail (Figure 5a and b). It is rather unusual that *Albino* RNAi did not lead to homeostasis defects even when the worms were continuously interfered with *Albino* dsRNA for more than 100 days. However, we did observe a mesenchymal and gut-like expression pattern of *ALAS* (Supplementary Figure S6B), and *Albino* RNAi did not result in a complete loss of the *ALAS* mRNA level (Supplementary Figure S4A). Thus, it is possible that *ALAS* is involved in additional processes other than *Albino*-mediated pigmentation and is required for the homeostasis of planarian independently of *Albino*. Although *PBGD*-interfered worms displayed no survival or regeneration abnormalities, they exhibited *Albino* RNAi-like albino phenotype at ~60 days post first RNAi (Figure 5b and c and Supplementary Figure S6A). Although *PBGD* RNAi caused a delayed phenotype emergence compared with *Albino* RNAi, worms that lost *PBGD* eventually became completely albino (Figure 5c). We thus used TEM to examine subcellular changes upon *PBGD* RNAi and detected similar pigment granules lost in *PBGD* RNAi worms (Figure 5d and Supplementary Figure S6C).

Gene functions of the remaining enzymes (for heme biosynthesis: *CPOX*, *PPOX*, *UROD* and *URO3S*; for ommochrome biosynthesis: *Afmid1* (*arylformamidase*), *Afmid2*, *Kmo1* and *Kmo2*) were detected (Supplementary Figure S6D and E). However, loss of any of these enzymes did not lead to pigmentation defects like *Albino* RNAi. Nonetheless, a slight body color alteration was observed under *Kmo2* RNAi, suggesting that ommochrome derived from tryptophan may partially be involved in the body color of planarian. Conversely, *Tyrosinase* (*tyr*), an enzyme critical for melanin biosynthesis, RNAi led to eyespot pigmentation failure only, rather than body pigment loss (Supplementary Figure S6F). These results revealed that, among the *Albino*-regulated tetrapyrrole biosynthesis enzymes, *PBGD* in particular is required for planarian body color maintenance, suggesting that *PBGD* serves as the downstream target of *Albino* in regulating the body pigment of planarian. Further validation with chromatin immunoprecipitation was required to demonstrate a direct regulation between *Albino* and *PBGD*. Moreover, we provide evidence that tetrapyrrole is involved as body pigment across evolution, demonstrating an exceptional evolutionary position of planarians.

### *PBGD* labels planarian pigment cells

**Figure 6:**
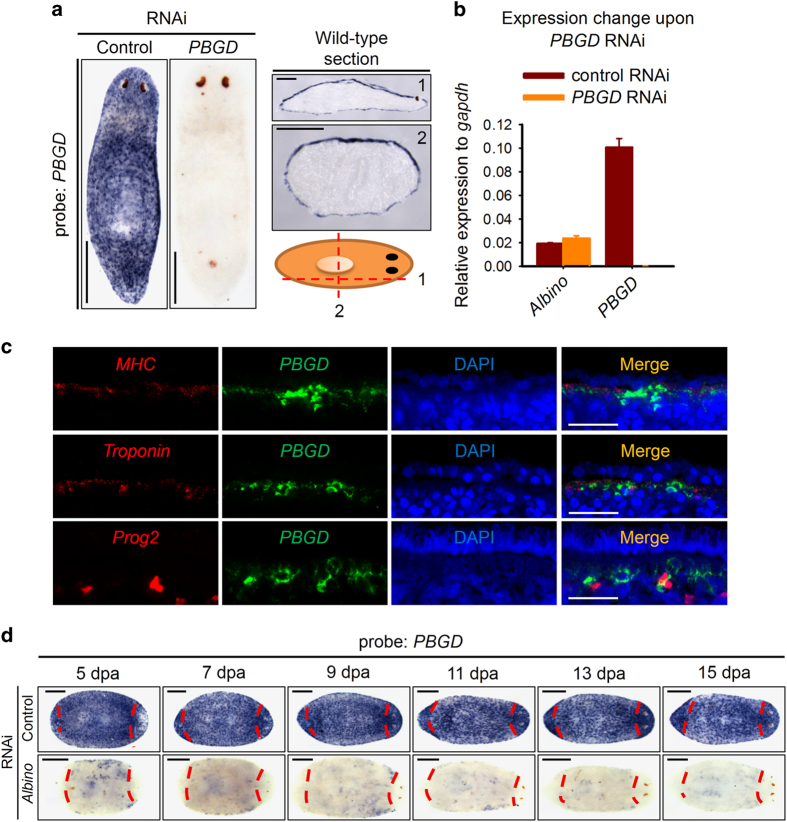
*PBGD* labels pigment cells in planarian. (**a**) WISH and frozen section of *PBGD* in wild-type animals showing an epidermal-specific expression pattern. Scale bar: 200 μm. (**b**) Relative expression level to *gapdh*. Shown are averages of three independent experiments; error bars=s.d. (**c**) Double FISH for *PBGD* with *mhc*, *troponin* and *prog2* in wild-type animals at dorsal body wall showing *PBGD*-positive cells lying in between muscle cells. Scale bar: 20 μm. Images are single confocal sections. (**d**) WISH showing expression patterns of *PBGD* in regenerating control or *Albino* RNAi worms. Red dashes indicate amputation sites. Scale bar: 200 μm.

We next confirmed the superficial expression of PBGD by whole-mount and cross-section ISH (Figure 6a). The expression of *PBGD* is efficiently knocked down without affecting *Albino* expression, suggesting that *Albino* functions upstream of *PBGD* (Figure 6a and b). We noticed that, unlike *Albino* or *ALAS*, *PBGD* expressed only at the superficial level, prompting us to investigate whether *PBGD* labels planarian pigment cells specifically. To gain insight into the accurate location at which *PBGD*-positive cells reside, we investigated the expression relationship between *PBGD* and stem cell progeny *prog2* and muscle cell markers *mhc* and *troponin*. We observed that *PBGD-*positive cells were situated between muscle cells just beneath the basal lamina (Figure 6c), a unique location for pigment cells (Figure 1d). Unlike *Albino*, *PBGD* is restricted within pigment cells without expressing in epidermal cells. Moreover, *PBGD* also enriched at newly regenerated blastema since 7 dpa (Figure 6d). The enrichment lasted for another 7 days during which planarians became pigmented (Figure 1a). Thus, the function and expression pattern of *PBGD* indicated that *PBGD* serves as a perfect molecular maker for labeling planarian pigment cells.

### *Albino* bridges between adult stem cells and *PBGD*-positive pigment cells

**Figure 7:**
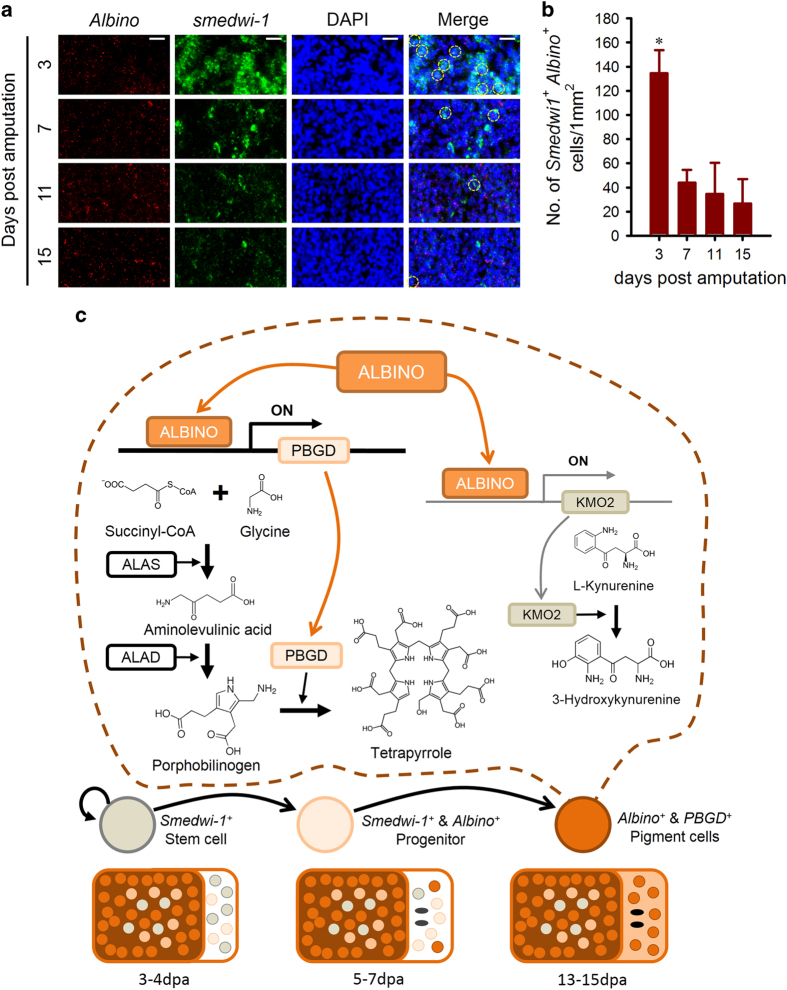
*Albino* mediates neoblast-pigment cell differentiation. (**a**) Double FISH for *smedwi-1* with *Albino* at different times of regeneration showing that *smedwi-1* and *Albino* colocalize at the blastema during regeneration. Scale bar: 20 μm. Yellow circles indicate double-positive cells, whereas red and green circles indicate *Albino* and *smedwi-1* single-positive cells, respectively. Images are single confocal sections. (**b**) Quantification of *smedwi-1* and *Albino* double-positive cells. Cells in 0.1 mm^2^ were counted in three independent experiments. Error bar=s.d.; *P<0.0001; significance determined with Student’s *t*-test. (**c**) Cartoon illustrates that *Albino* controls the expression of *PBGD,* and *smedwi-1*-positive neoblasts initially specialize into *smedwi-1* and *Albino* double-positive cells and then fully differentiate into pigment cells expressing *Albino* and *PBGD*. During regeneration, *Albino*-expressing neoblasts accumulate in the blastema at 3 dpa and initiate the expression of tetrapyrrole biogenesis enzymes. The *Albino* and *PBGD* double-positive cells accumulate within blastema since 7 dpa, and finally these cells generate tetrapyrroles that get the planarians pigmented.

RNAi of *Albino* not only sabotaged the pre-existing *Albino* expression but also blocked the enrichment of *PBGD* at blastema during regeneration (Figure 6d). We next studied the expression dynamics of *Albino* together with *smedwi-1* and *PBGD* during regeneration. DFISH showed at 3 dpa that a significant number of *smedwi-1* and *Albino* double-positive cells emerged at the blastema and, as regeneration proceeded, the number of *smedwi-1* and *Albino* double-positive cells declined to a relatively stable level (Figure 7a and b and Supplementary Figure S7A), suggesting that the fate of most pigment cells was determined right after the local neoblast proliferation [47]. However, the *Albino* and *PBGD* double-positive cells did not begin to concentrate at blastema until 7 dpa and enrichments of *Albino* and *PBGD* double-positive cells last until the pigmentation of blastema is finished (Supplementary Figure S7B), suggesting that *Albino* has a regulatory role during the fate decision of the stem cell subpopulation that differentiate into pigment cells.

Our data thus suggest that, during the pigmentation of newly regenerated blastema, a subpopulation of *smedwi-1*-positive stem cells express *Albino* and, under the control of *Albino*, tetrapyrrole biosynthesis enzymes begin to express and synthesize tetrapyrrole as the body pigment of planarians. Meanwhile, the expression of *Kmo2*, a key enzyme in ommochrome biosynthesis, also depends on *Albino* (Figure 7c).

## Discussion

Dysregulation of pigmentation process leads to serious disorders in humans [48]. We set up a planarian pigmentation model to search for *de novo* mechanisms during the differentiation from stem cells to pigment cells. In this work, we identified a forkhead domain containing transcription factor *Albino* required for planarian body color pigmentation. We elucidated that transcription factor *Albino* is required for the expression of *ALAS*, *ALAD* and *PBGD* expression, thereby controling the tetrapyrrole biosynthesis. Interestingly, apart from the tetrapyrrole biosynthesis pathway, the expression of *Kmo2*, a key enzyme in the ommochrome pathway, also depends on *Albino*, suggesting that *Albino* controls two pigment biosynthesis pathways within planarian body pigment cells (Figure 7c). Meanwhile, our results revealed that *PBGD* is specifically expressed within planarian body pigment cells, thus providing the best marker that labels planarian body pigment cells. Besides, the regulatory relationship between Albino and tetrapyrrole enzymes suggests new prospects in porphyria pathogenesis study.

### The role of *Albino* in adult stem cell and other non-pigment-related cells

Our result demonstrated that *Albino* has a critical role in regulating tetrapyrrole biosynthetic enzyme expression that takes place in pigment cells. However, the role of *Albino* in adult stem cell and other non-pigment-related cells remains unclear. The neoblasts that participated in regeneration in planarian are heterogeneous, consisting of pluripotent stem cells and lineage-committed progenitors [49]. Transcription factors have critical roles in guiding the specification processes [8, 9]. According to our ISH results, *Albino* is broadly expressed, but *Albino* RNAi showed no other defects but in pigmentation. We assume that *Albino* functions in a modest manner in non-pigment cells without affecting the cell survival or other biological processes. However, as *Albino* is not completely knocked out, it is possible that the RNAi efficiency is critical for *Albino* functions. An *Albino* knockout strain will be of great help in understanding the function of this transcription factor. Nonetheless, we provided planarian as an alternative model for pigmentation study, as markers labeling early and late differentiated pigment cells are available. By monitoring *Albino* expression, we could observe the early specialization from neoblasts to pigment cells under various stimuli, such as light bleaching.

### *Albino* simultaneously controls two pigment biosynthesis pathways

Our study presents the link between forkhead genes and pigmentary system development by demonstrating that *Albino* controls the expressions of tetrapyrrole biosynthetic enzymes that in turn generate tetrapyrrole. *Albino* RNAi resulted in over 100-fold decrease in *PBGD* expression, suggesting a direct regulatory relationship between *Albino* and *PBGD*. We provided evidence that tetrapyrroles are used as body pigment in planarian across evolution, suggesting a unique evolutionary position of planarians. However, as we failed in generating a planarian-specific Albino antibody, we could not elucidate the direct regulatory mechanism. A functional Albino antibody is urgently in need for further analyses. We also noticed a significant expression reduction in *Kmo2*, a critical enzyme in ommochrome generation, upon *Albino* RNAi. Although only a very weak depigmentation was observed upon *Kmo2* RNAi, we revealed a similar expression pattern between *Kmo2* and tetrapyrrole enzymes, suggesting that *Albino* simultaneously controls two pigment biosynthesis pathways.

### Identification of planarian pigment cell markers provides a new model for pigmentation study

Expression analysis of *PBGD* showed a specific expression within the muscle cells around the surface of planarians. In addition, the shape of *PBGD*-positive cells resembles the shape of pigment cells under the observation of TEM. Meanwhile, RNAi of *PBGD* led to complete albinism, although it took more time than *Albino* RNAi. We conclude from the expression pattern and RNAi phenotype that *PBGD*-positive cells represent planarian pigment cells and thus provide a molecular marker for pigment cells of planarians. Meanwhile, as *Kmo2* RNAi showed a rather weak body color reduction (Supplementary Figure S6E), which is supported by the recently published paper [46], thus *PBGD* is a better marker for labeling planarian pigment cells.

Several *in vivo* and *in vitro* models have been established for pigmentation study, especially for melanocyte studies [50, 51]. However, thus far, the complexity of the vertebrate pigmentary system made it difficult to observe pigmentation and depigmentation dynamically. Stubenhaus *et al.* [46] recently suggested that depigmentation induced by intense light in planarian models the pathophysiology of acute porphyrias. Our results elaborated *Albino* as the upstream regulator of two evolutionarily conserved planarian body pigment synthesis pathways, and this warrants further investigation on whether *Albino* has a role in the pathophysiology of acute porphyrias. We have shown that planarian lost body color upon *Albino* or *PBGD* dsRNA feeding. This depigmentation process provides a new *in vivo* animal model to study the biological alteration upon pigment loss. Meanwhile, when dsRNA foods were substituted with normal foods, planarians became re-pigmented under the effects of unaffected and pluripotent cNeoblasts system [7]. The re-pigmentation process provides a controllable pigmentation model from specialized stem cells. Our model provides the basis for elucidating the essential role of *Albino* in the stem cell-pigment cell fate decision.

## Materials and Methods

### Planarian culture

Clonal lines of hermaphroditic and asexual (CIW4) *S. mediterranea* were maintained as previously described [52] and supplied with 0.21 g l^−1^ Instant Ocean salts. Animals were fed weekly with homogenized calf liver. Animals were starved for 1 week before any experiments. For irradiation, planarians were exposed to 100 Gray of gamma irradiation using a sealed source of Cesium 137 (Gammacell3000, MDS Dordion, Chalk River, ON, Canada). The animals were kindly provided by P Newmark (University of Illinois at Urbana-Champaign/Howard Hughes Medical Institute, Urbana, IL, USA), P Reddien (Massachusetts Institute of Technology/Howard Hughes Medical Institute, Cambridge, MA, USA) and N Oviedo (University of California, Merced, CA, USA).

### RNAi experiments

We use *in vitro* synthesized dsRNA for RNAi experiments. Each time, we prepared 400 ng dsRNA mixed with 5 µl liver for each worm and the total volume depends on the number of worms. The worms were fed four times in screening, *Albino*, *ALAD* and *PBGD* RNAi, two times for *smedwi-2*, *ALAS* and *UROD* RNAi, eight times for *CPOX*, *PPOX*, *URO3S*, *Afmid1* and/or *Afmid2Kmo1* and/or *Kmo2* RNAi. We fed worms for 12 times in survival experiments of *Albino*, *ALAD* and *PBGD* RNAi. We fed the worms on days 1, 4, 7 and 10 for the first four times and fed the worms every 10 days with liver mixed with corresponding dsRNA. We observed the successful food uptake for every single experiment. At least 10 worms were used in each RNAi experiment and at least three independent experiments were carried out for each gene. Phenotypes shown in all replicates are presented; otherwise the specific number was labeled.

### Gene cloning

In the transcription factor screen, we used a BLAST-based reciprocal best-hit method, in combination with protein sequence alignment and phylogenetic analysis as described previously. We searched for forkhead containing proteins in both planarian genome database SmedGD (http://smedgd.neuro.utah.edu), the Plan Mine (http://planmine.mpi-cbg.de) and the hermaphroditic strain (Expressed Sequence Tag) database [53–55]. We obtained the full-length sequences with the RNA ligase-mediated rapid amplification of cDNA ends kit (Ambion, Austin, TX, USA) and aligned with online PFAM (http://pfam.xfam.org/) and local ClustalX2. Phylogenetic trees were constructed with ClustalX2 using the neighbor-joining algorithm with 1000 trials of bootstrap and 120 random seeds. All sequences were deposited in GenBank.

### Transmission electron microscopy

TEM was performed as previously described [35]. In brief, worms were first fixed with primary fixative. After being washed with EM buffer, secondary fixation was performed with osmium tetroxide. Ultrathin sections were stained with uranyl acetate and lead citrate, and were observed with a TEM (H-7650; Hitachi High Technologies America, Inc., Pleasanton, CA, USA).

### Whole mount in situ hybridization and Fluorescence in situ hybridization

Whole mount in situ hybridization (WISH) and FISH were performed as previously described [56, 57]. In brief, worms were killed in 5% n-acetyl cysteine (Sigma-Aldrich, St Louis, MO, USA), fixed in 4% paraformaldehyde (PFA), permeabilized using reduction buffer and dehydrated in a graded series of methanol in PBSTx before bleaching. After rehydration, hybridizations were performed with 0.1~0.5 ng per ulriboprobes. For WISH, we use anti-digoxigenin-alkaline phosphatase, 1:4 000 (Roche, Pleasanton, CA, USA). Signal was developed using nitro-blue tetrazolium chloride (NBT)/5-bromo-4-chloro-3'-indolyphosphate p-toluidine salt (BCIP) substrate (1:50; Roche). For FISH or double FISH, we first generated fluorescein isothiocyanate (FITC)-tyramide and rhodamine-tyramide as previously described [58] by using fluorescein mono-n-hydroxysuccinimide-ester (46410; Pierce, Waltham, MA, USA), rhodamine mono-n-hydroxysuccinimide-ester (46406; Pierce) and tyramide (T-2879; Sigma-Aldrich). For double FISH, peroxidase (POD) antibodies (11207733910, 11426346910, Roche) were used in 1:500 and inactivation was performed with 4% PFA for 60 min. Within a given experiment, all samples were developed in the fluorescent substrate for the same length of time and imaged using identical exposure conditions. All sections were performed post WISH or FISH. Frozen sections was performed as previously described [59] with modifications. In brief, FISH-stained animals were transferred to a graded series of sucrose in PBS before embedding in optimum cutting temperature compound (Sakura, Torrance, CA, USA). Specimens were frozen in liquid nitrogen and were sectioned in a cryostat (Microm HM550; Thermo Fisher Scientific, Waltham, MA, USA) at 16 µm at −20 °C. Vibration sections were made to obtain thicker sections. Specimens were embedded with low-melting-point agarose and then the sections were performed with Automated Vibratome (VT1200 S, Leica, Wetzlar, Germany) at 80 µm. The sections were placed on charged slides (Premiere, Shanghai, China) and mounted with Mowiol mounting medium before imaging.

### Image acquisition, processing and quantification

Live animals, WISH samples mounted with Mowiol mounting medium, were photographed using a microscope (SteREODiscovery.V20; Carl Zeiss, Jena, Germany) equipped with a Plan Apochromat ×1.0 objective and a digital microscope camera (AxioCamHRc; Carl Zeiss) automated using the AxioVision Rel.4.8 software (Carl Zeiss). FISH specimens were mounted with fluorescence mounting medium (Dako, Glostrup, Denmark) or Mowiol mounting medium, and the images were captured with a laser-scanning confocal microscope (True Confocal Scanner SP5; Leica; HCX Plan Apochromat confocal scanning ×10/0.4 NA, ×20/0.7 NA, ×40/0.85 NA or ×63/1.40 NA oil immersion objective lens) using the LAS AF software (Leica). Images were processed with the LAS AF Lite software and Photoshop software (Adobe, San Jose, CA, USA) and were quantified using the QWin software (Leica) or the ImageJ software (National Institutes of Health, Bethesda, MD, USA). All ISH experiments were performed, imaged and processed identically (at room temperature, 22 °C) to allow direct comparison between experimental animals and controls.

### RNA extraction, qPCR and gene expression profiling

qRT-PCR was performed as previously described [60]. In brief, total RNA was isolated using TRIZOL (Invitrogen, Waltham, MA, USA). cDNAs were generated from 300 to 500 ng of total RNA with the FastQuant RT Kit with gDNAse (Tiangen, Beijing, China). Gene-specific primers were designed with Oligo Perfect designer (Invitrogen). qPCRs were performed with the Ace Q qPCR SYBR Green Master Mix Kit (Vazyme, Nanjing, China). At least three biological replicates were performed, and each experiment was performed with triplicate or quadruplicate PCR reactions. Data are expressed using the comparative cycle threshold method. Relative expression levels were normalized to the levels of GAPDH (AY068133) mRNA and plotted with SigmaPlot 11.0 (Systat Software Inc., San Jose, CA, USA) Gene arrays applied the Agilent Custom array described previously [6]. The RNAi worms were fed with dsRNA for four times and RNA samples were collected using TRIZOL (Invitrogen) 7 days post the last feeding. Those genes with a decrease in expression of upto twofolds in both *Albino* RNAi groups were selected (Table 1). Genes with significant changes upon Albino RNAi were attached in Supplementary Table S1 ‘Differential expressed genes in microarray and gene information’.

### Light-bleaching experiments

Worms were placed under direct light for 9 days. An 11-watt fluorescent lamp was placed 15 cm above the 10 cm dishes. The illuminance was 3 000 lux detected using digital light meter (TES Electrical Electronic Corp., Taipei, Taiwan). Each dish contains 10 or less worms and the culture water was replaced every day.

### Flow cytometry

Sorting by flow cytometry was performed as previously described [61, 62]. In brief, planarians were diced into small pieces on a cold plate and incubated in 1 mg ml^−1^ collagenase (diluted in calcium- and magnesium-free medium plus 1% bovine serum albumin) as previously described. Dissociated cells were filtered with a cell strainer (BD, Franklin Lakes, NJ, USA) and stained with 0.2 μg ml^−1^ calcein acetoxymethylester and 18 μg ml^−1^ Hoechst 33342 for an appropriate time. After incubating with 5 μg ml^−1^ propidium iodide, analyses and sorts were performed using the FACSAria II (BD) or MoFlo XDP (Beckman Coulter, Brea, CA, USA). Data were processed using FlowJo V7.6.5 (Tree Star Inc., Ashland, OR, USA).

### Statistical analysis

Results are presented as means±s.d., and statistical analyses were performed in SigmaPlot 11.0 using the Student’s *t*-test for two groups or one-way analysis of variance for three or more groups. *P*<0.05 was considered significant.

## Figures and Tables

**Table 1:** List of downregulated genes upon Albino RNAi in array analysis

*Probe*	*Fold change*	*BLAST*	*GI number*
09827	0.01	Porphobilinogen deaminase isoform 1	84609767
00920	0.04	Threonine dehydratase catabolic	113467172
07123	0.05	Saposin	113466959
07980	0.11	5-Aminolevulinic acid synthase	84613222
04488	0.13	Synergin gamma	84612706
01820	0.13	Zinc-finger protein 474	84600384
03955	0.14	Carbonic anhydrase	84614305
01039	0.17	Fucolectin	84599334
05195	0.18	Ferritin	84598371
06678	0.19	Lipase	84592609
06806	0.20	Elav1 (HUR)	84613280
02989	0.21	Y box protein 4-like protein	84613514
02568	0.22	Hexokinase	84591284
00559	0.23	C3H-zinc-finger-containing protein 1	84600517
06162	0.23	Granulin-like protein	84613008
02604	0.24	Ferritin	84596222
00736	0.25	Lipase	84610165
01255	0.29	Serine protease inhibitor-1	84601442
01819	0.31	Trans-1,2-dihydrobenzene-1,2-diol dehydrogenase	84597330
03500	0.37	Sodium-dependent glucose transporter 1	84613285
09907	0.37	Delta-aminolevulinic acid dehydratase	116034941
03741	0.41	Lectin 1	84597836
00821	0.42	Lipase	84598548

Abbreviation: BLAST, The Basic Local Alignment Search Tool; GI, GenInfo Identifier; RNAi, RNA interference.

Fold changes were average from two replicas.
