# Supplementary material for: Forkhead containing transcription factor Albino controls tetrapyrrole-based body pigmentation in planarian
Source: Cell Discov. 2016 Aug 2;2:16029–. doi: 10.1038/celldisc.2016.29 (PMC4969599; doi:10.1038/celldisc.2016.29)
Supplement: Supplementary Figure Legend [file celldisc201629-s1.doc]

**Supplemental Figure 1. Illustration of planarian pigmentary system.**

(a) The pigmentation process of worms 2-12 days post hatching. Lower panels are magnified views of yellow dash frame areas. Scale bar: 200 µm. The arrows indicates newly generated pigments. (b) Transmission electron micrographs of the area that pigment cells reside. LM, longitudinal muscle.CM, circular muscle. Scale bar: 20µm. Yellow arrows indicate pigment granules. (c) The pigmentation process of regenerating worms during 2-16 days of regeneration. White dashes indicate the amputation sites. Scale bar: 200 µm. (d) Cartoon that illustrate the position that planarian pigment cells reside. LM, longitudinal muscle.CM, circular muscle. (e) TF_FK_025 is clustered with human FOXP family proteins. (f) Conserved domain analysis revealed a Forkhead domain within protein TF_FK_061 and TF_FK_062.

**Supplemental Figure 2. *Albino* RNAi leads to albinism in planarian.**

(a) Planarian upon *Albino* RNAi gradually lost body pigments. Scale bar: 200 µm. (b) Transmission electron micrographs showing the pigment lost in *Albino* RNAi worms. Scale bar: 50 µm.

**Supplemental Figure 3. *Albino* expresses at epidermal region and a subpopulation of neoblasts.**

(a) Double *FISH* with *prog2* and *Albino* in wild type animal showing the dorsal body wall. Scale bar: 20 µm. Images are single confocal sections. (b) Expression distribution of *Albino* in FACS sorted cells. (c) WISH in wild-type and γ-ray irradiated animal for *Albino*. Scale bar: 500 µm. (d) WISH in 2 days post hatching and mature animal for *Albino*. Scale bar: 500 µm.

**Supplemental Figure 4. Planarian tetrapyrroles biosynthesis enzymes are evolutionary conserved.**

(a) Phylogenetic tree showing that planarian *ALAD*, *ALAS* and *PBGD* are clustered into their own family respectively. Human, mouse and fruit fly were used as reference species. (b) Amino acids identities of ALAD, ALAS and PBGD with human and mouse. (c) ClustalX2 alignment results showing PBGD is highly conserve with human and mouse.

**Supplemental Figure 5. Expression changes of heme, ommochrome and melanin biogenesis enzymes upon *Albino* RNAi.**

(a) Expression folds changes of heme biosynthetic enzymes (post tetrapyrroles) upon *Albino* RNAi. Shown are averages of three independent experiments; error bars= SD. (b) WISH for heme biosynthetic enzymes (post tetrapyrroles) in worms upon control or *Albino* RNAi. Scale bar: 500 µm. (c) Expression folds changes of melanin and ommochrome biosynthetic enzymes upon *Albino* RNAi. Shown are averages of three independent experiments; error bars= SD. (d) WISH for *Kmo2* in worms upon control or *Albino* RNAi. Scale bar: 500 µm. (e) Representative double FISH results of *kmo2* with *Albino* in wild type animal. Images are single confocal sections. (f) Wild type worms and worms received 9 days of continuous direct light. Scale bar: 500 µm.

**Supplemental Figure 6. RNAi of heme, ommochrome and melanin biogenesis enzymes.**

(a) *ALAD* and *PBGD* RNAi worms successfully regenerated missing heads and tails. Scale bar: 200µm. (b) Frozen section of *ALAS* in wild type animal showing a mesenchymal and gut like expression pattern. Scale bar: 200µm. (c) Transmission electron micrographs showing the pigment lost in *PBGD* RNAi worms. Scale bar: 50 µm. (d) RNAi of heme biosynthetic enzymes (post tetrapyrroles). Scale bar: 200µm. (e) RNAi of ommochrome biosynthetic enzymes. Scale bar: 200µm. (f) RNAi of *Tyrosinase*. Scale bar: 200µm.

**Supplemental Figure 7. *Smedwi-1* with *Albino* double positive cells enriches at 3dpa.**

(a) Double FISH for *smedwi-1* with *Albino* at different times of regeneration showing *smedwi-1* and *Albino* colocalize at the blastema during regeneration. Scale bar: 20 µm. Yellow frames indicate zoom in zone showed in Figure 7a. Images are single confocal sections.

(b) Double FISH for *PBGD* with *Albino* at different times of regeneration. Scale bar: 20 µm. Yellow dashes indicated cutting sites. Images are single confocal sections.
