# Supplementary figures and images for: Forkhead containing transcription factor Albino controls tetrapyrrole-based body pigmentation in planarian
Source: Cell Discov. 2016 Aug 2;2:16029–. doi: 10.1038/celldisc.2016.29 (PMC4969599; doi:10.1038/celldisc.2016.29)

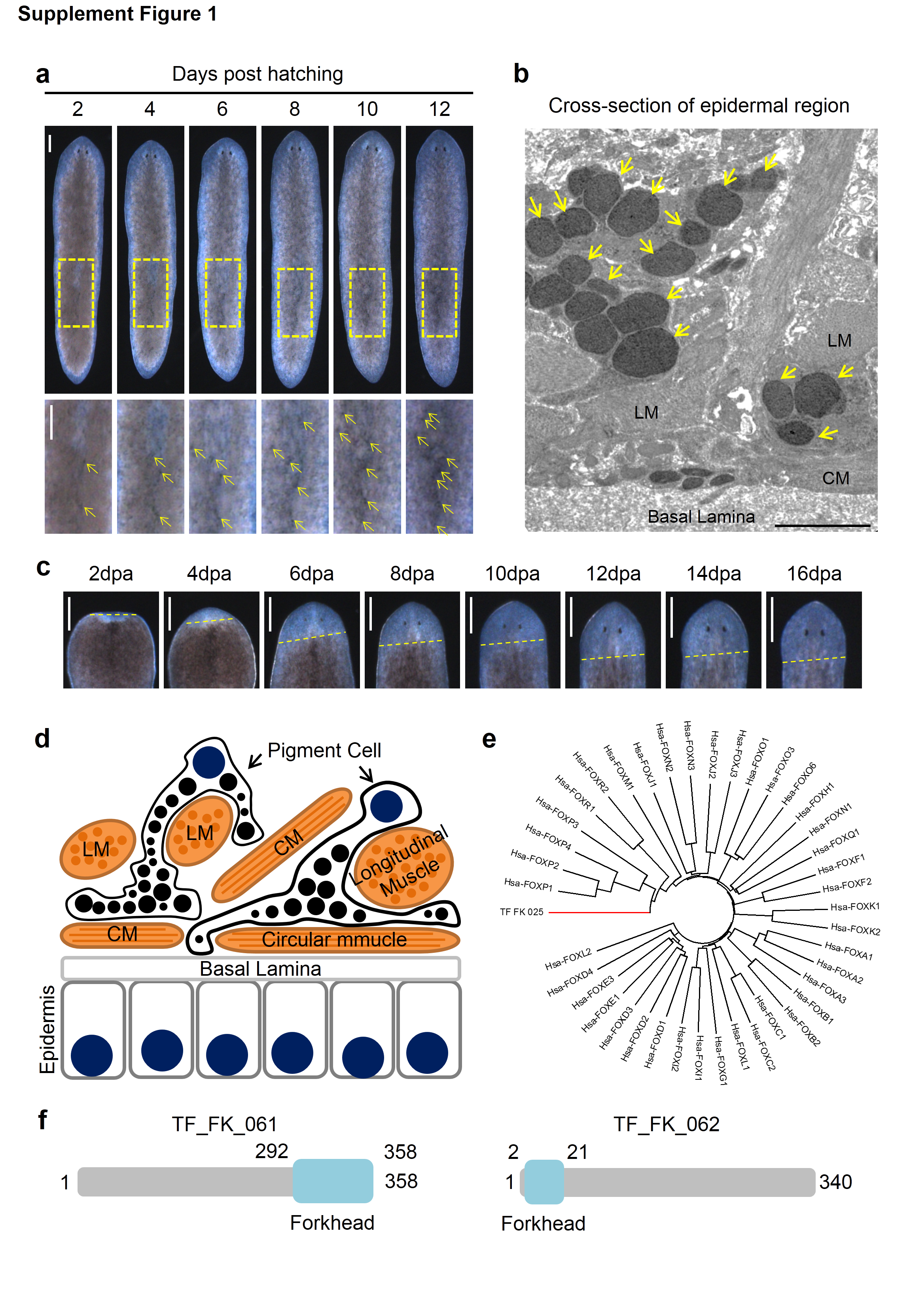

Supplement: Supplementary Figure S1 [file celldisc201629-s3.tiff]

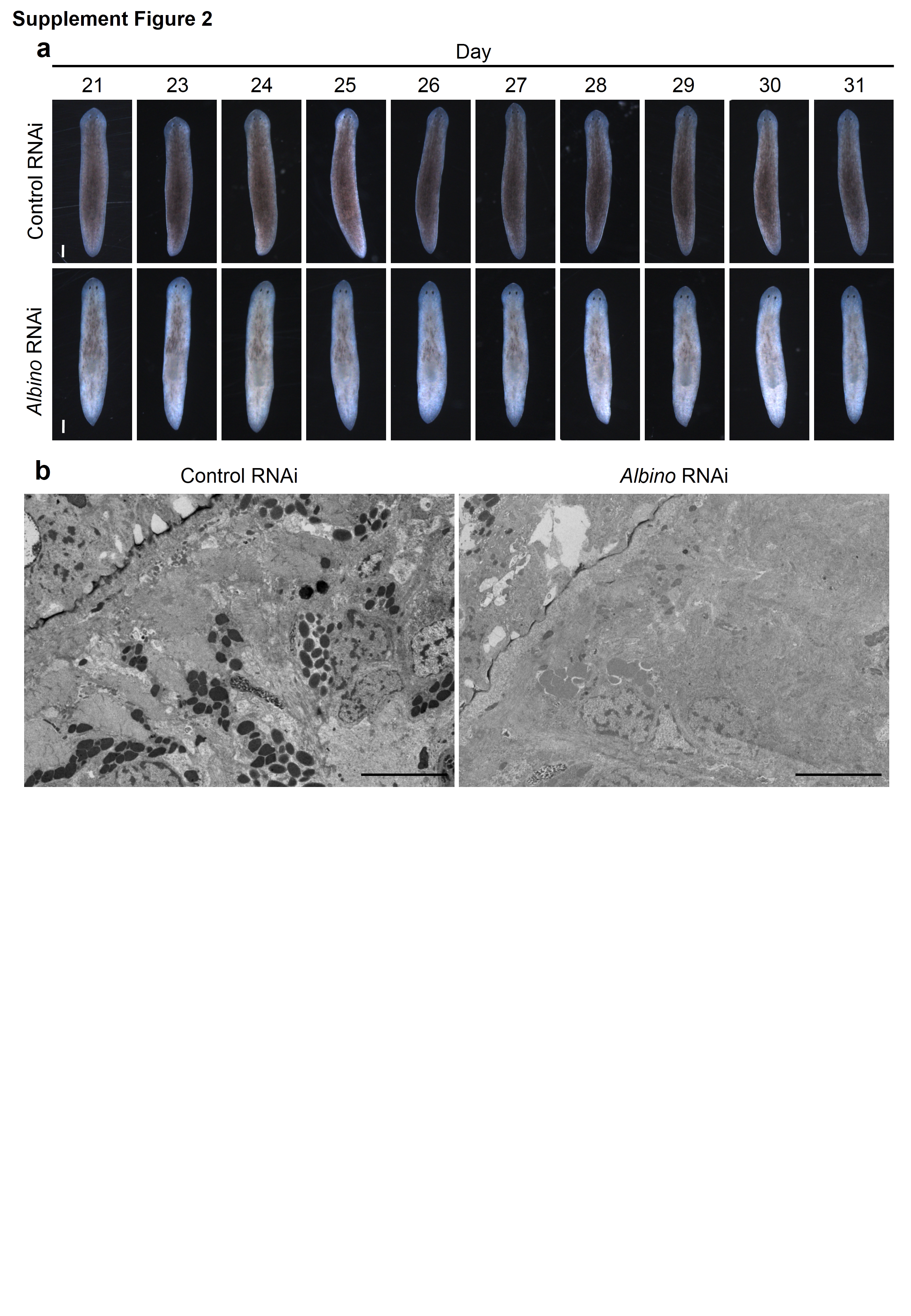

Supplement: Supplementary Figure S2 [file celldisc201629-s4.tiff]

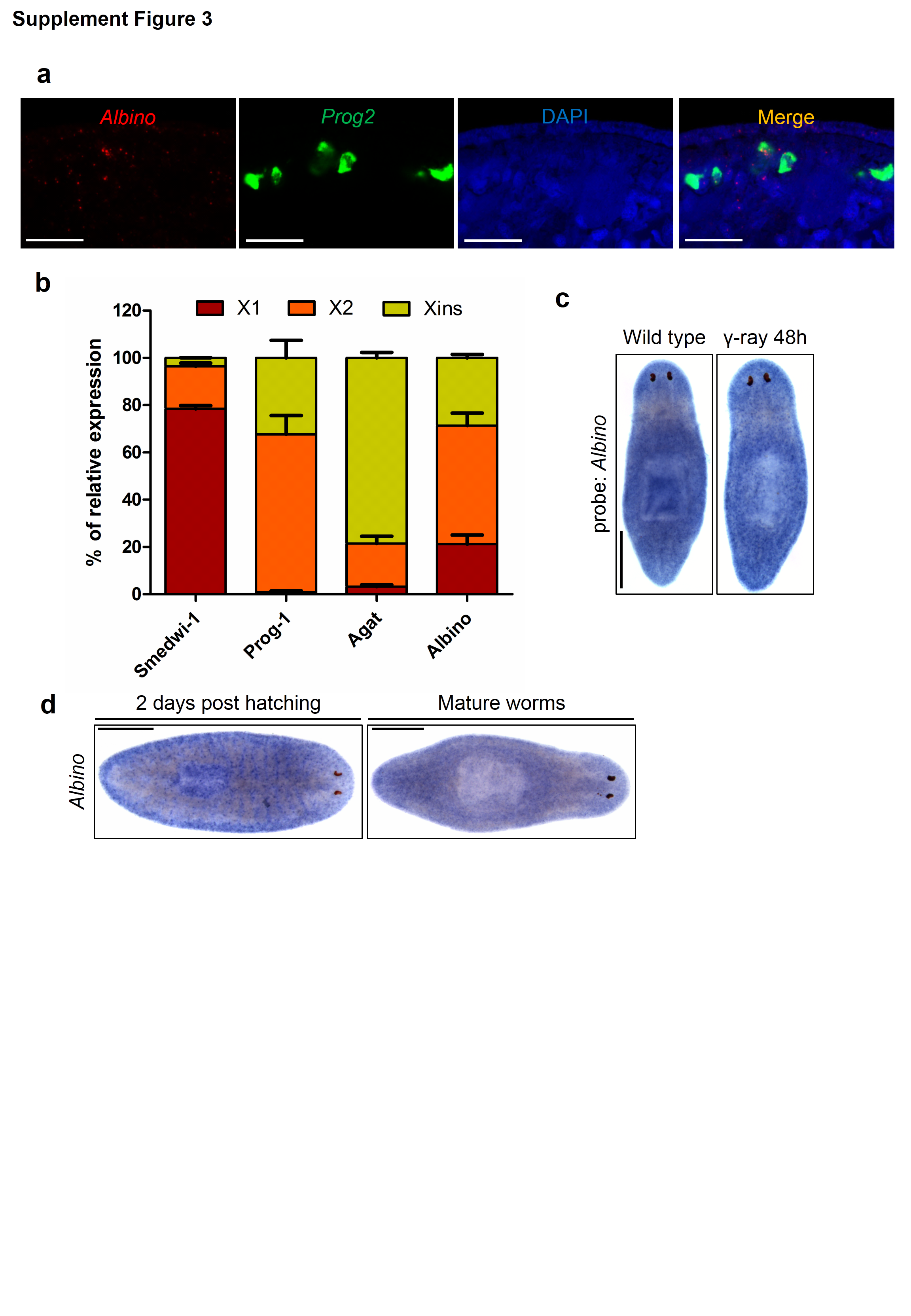

Supplement: Supplementary Figure S3 [file celldisc201629-s5.tiff]

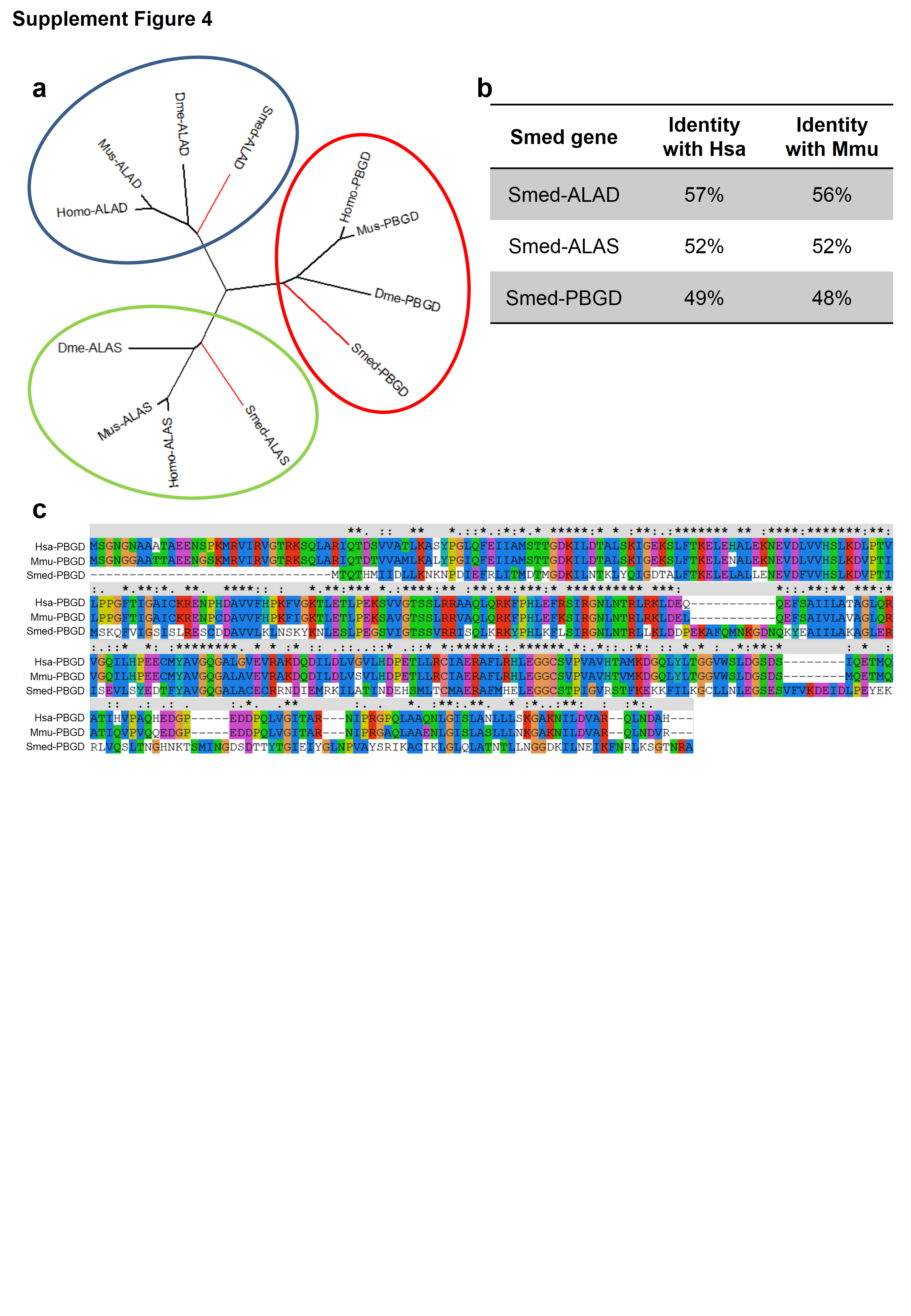

Supplement: Supplementary Figure S4 [file celldisc201629-s6.tiff]

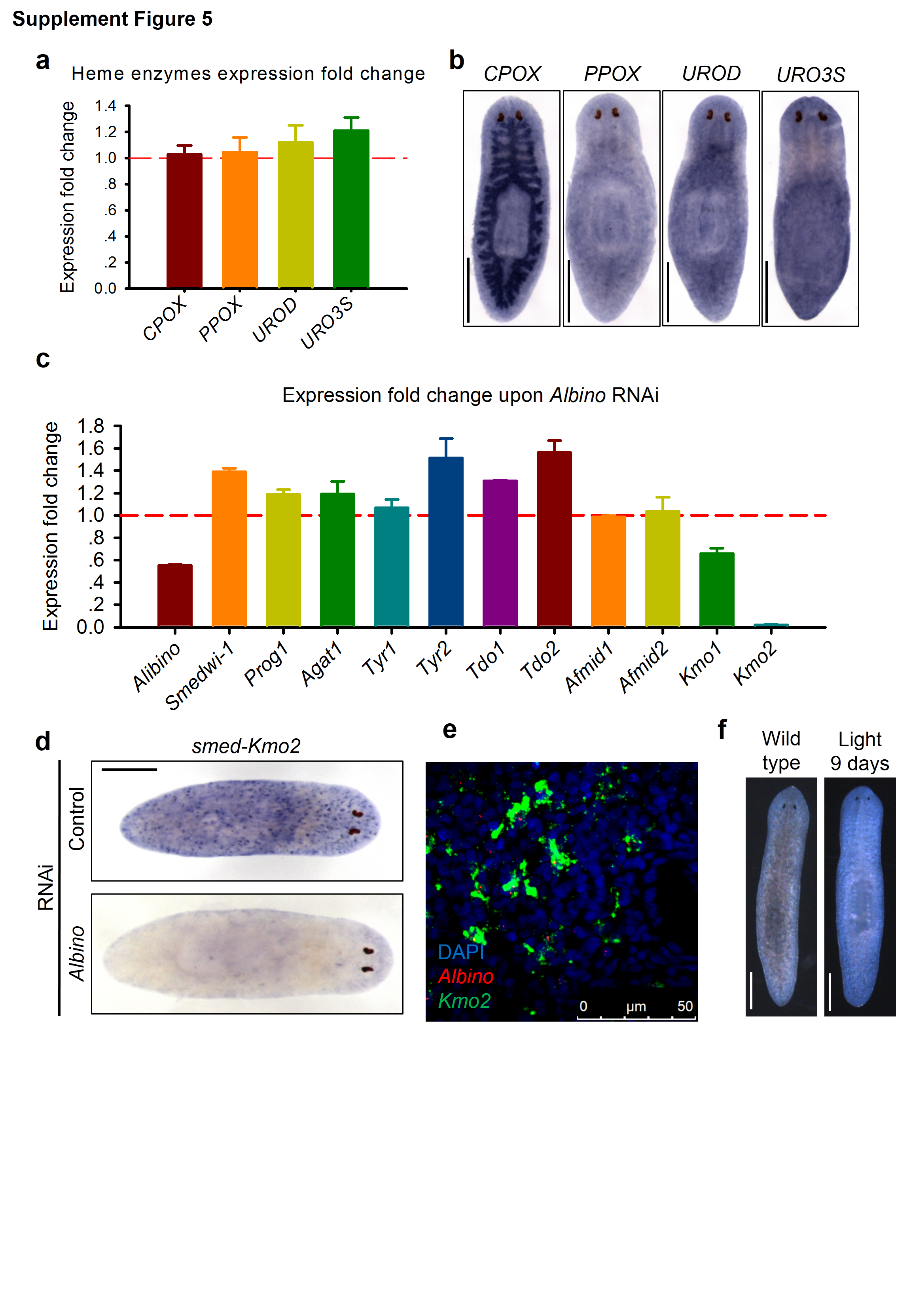

Supplement: Supplementary Figure S5 [file celldisc201629-s7.tiff]

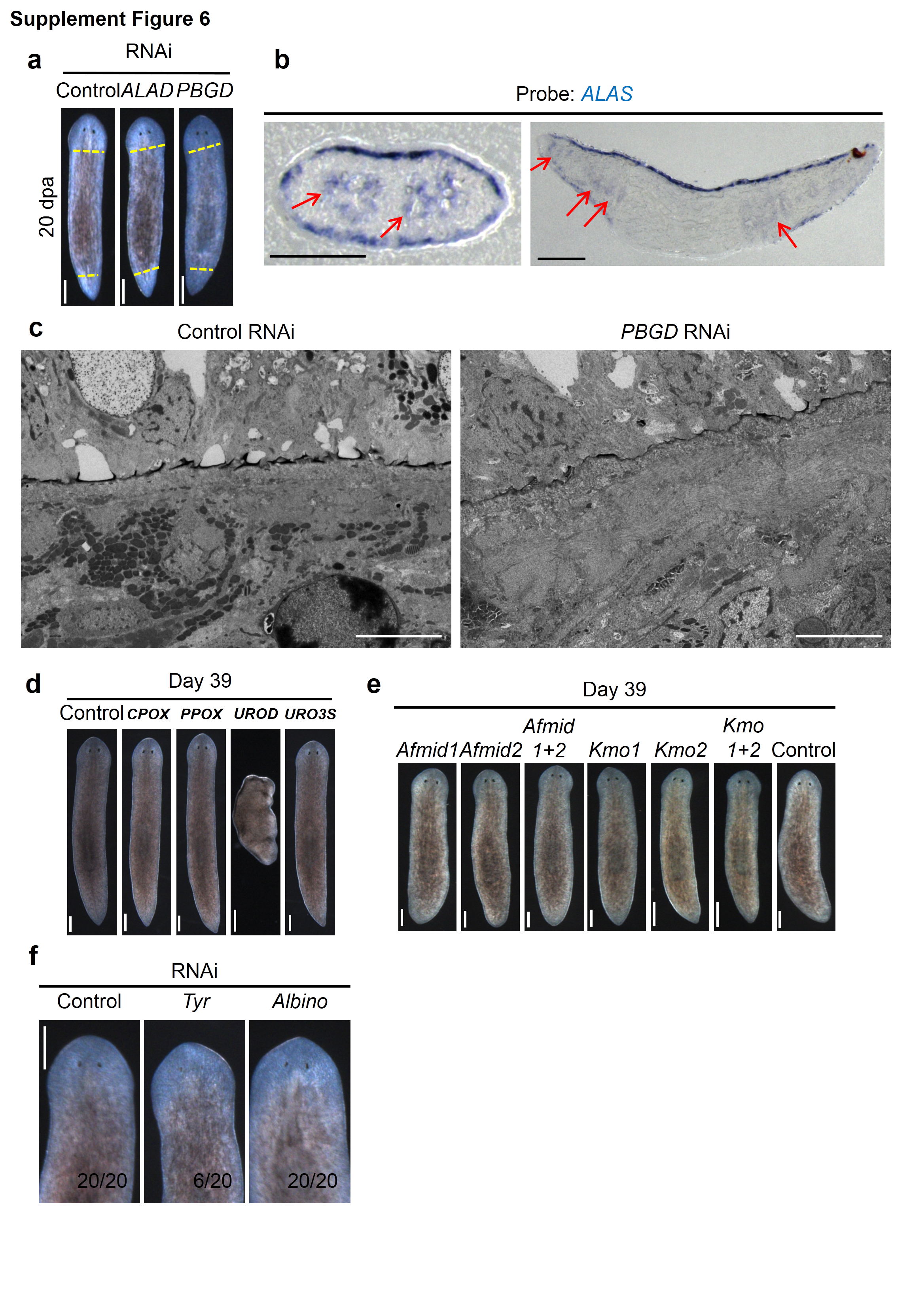

Supplement: Supplementary Figure S6 [file celldisc201629-s8.tiff]

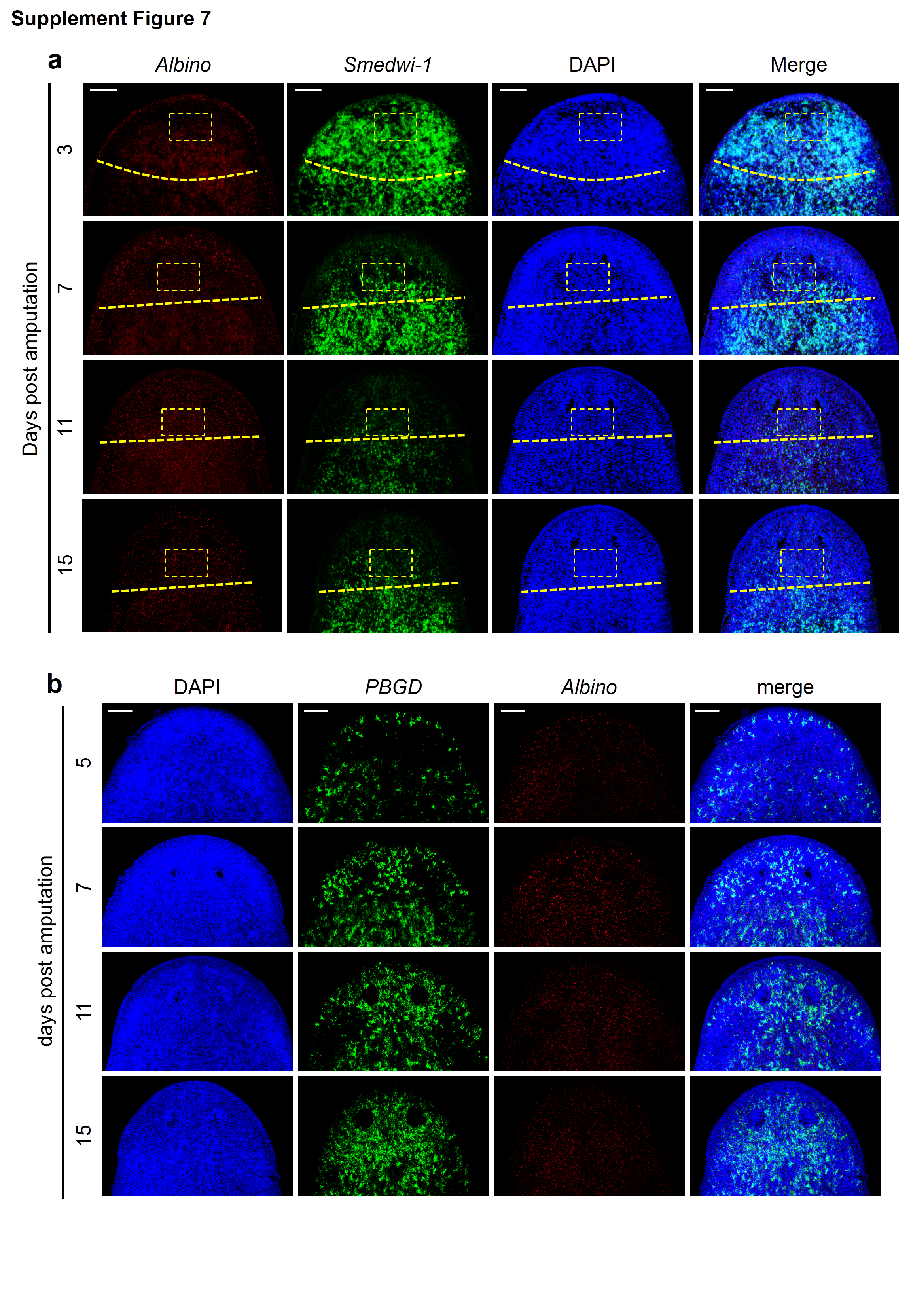

Supplement: Supplementary Figure S7 [file celldisc201629-s9.tiff]
